# Tumorigenesis role and clinical significance of DJ-1, a negative regulator of PTEN, in supraglottic squamous cell carcinoma

**DOI:** 10.1186/1756-9966-31-94

**Published:** 2012-11-14

**Authors:** Xiao-Lin Zhu, Zhang-Feng Wang, Wen-Bin Lei, Hui-Wen Zhuang, Wei-Jian Hou, Yi-Hui Wen, Wei-Ping Wen

**Affiliations:** 1 Department of Otorhinolaryngology Head and Neck surgery, The First Affiliated Hospital, Sun Yat-Sen University, 2nd Zhongshan Road 58#, Guangzhou, 510080, Guangdong, P.R. China; 2 Otorhinolaryngology Institute, Sun Yat-Sen University, 2nd Zhongshan Road 58#, Guangzhou, 510080, Guangdong, P.R. China

**Keywords:** DJ-1, PTEN, Tumorigenesis, Supraglottic squamous cell carcinoma, Overall survival

## Abstract

**Background:**

DJ-1 can induce the tumor cell proliferation and invasion via down-regulating PTEN in many malignant tumors, and correlated to prognostic significance. However, the tumorigenesis role and clinical significance of DJ-1 in supraglottic squamous cell carcinoma (SSCC) is unclear. We aimed to evaluate the DJ-1 the relationship between DJ-1 and clinicopathological data including patient survival.

**Methods:**

The expression of DJ-1 and PTEN in SSCCs (52) and adjacent non-cancerous tissues (42) was assessed by immunohistochemistry (IHC), and the relationship between DJ-1 and clinicopathological data was analyzed.

**Results:**

DJ-1 was detected mainly in SSCCs (88.5%) and less frequently in adjacent non-cancerous tissues (21.0%). PTEN expression was detected in 46.2% of SSCCs and in 90.5% of adjacent non-cancerous tissues. DJ-1 expression was linked to nodal status (P = 0.009), a highly significant association of DJ-1 expression with shortened patient overall survival (5-year survival rate 88.0% versus 53.9%; *P* = 0.007; log rank test) was demonstrated.

**Conclusions:**

Our data suggested that DJ-1 over-expression was linked to nodal status, and might be an independent prognostic marker for patients with SSCC.

## Background

Laryngeal squamous cell carcinoma (LSCC), one of the most common malignancies of the head and neck region, accounts for approximately 2.4% of new malignancies worldwide every year [1,2]. Supraglottic squamous cell carcinoma (SSCC), one advanced type of LSCC, is often accompanied by lymph node metastasis or even systemic metastasis, and usually results in substantial annual morbidity and mortality. Hence, to predict the biology of the tumor and the course of the disease in individual patient is importance for appropriate therapy and patient surveillance. The evaluation of a SSCC patient’s prognosis and predictive markers is primarily based on the clinical tumor-node-metastasis (TNM) staging [3]. However, patients with SSCC with similar clinical stage classifications usually have different clinical outcomes, suggesting that TNM staging is not sufficient for precisely determining a SSCC prognosis. Therefore, identifying specific biomarkers which have diagnostic and prognostic value for SSCC remains a priority.

DJ-1, a mitogendependent oncogene, was firstly reported by Nagakubo in 1997 [4]. Recent studies indicated that DJ-1 is closely related to the proliferation, metastasis, occurrence, and prognosis of the malignant tumors [2,5-13]. In our recent study of glottic squamous cell carcinoma [2], DJ-1 was shown as an independent molecular marker for poor prognosis, and was correlated with pT status and tumor grading. In other LSCC studies [2], DJ-1was also identified as an activator of cell proliferation, and was related to T stage and poor prognosis [14,15]. However, the relationship between DJ-1 and lymph node metastasis of LSCC have not been revealed both in our and others’ studies.

Phosphatase and tensin homologue (PTEN) is a dual-specific phosphatase that plays an important role in tumorigenesis and reduced PTEN expression is associated with cell survival, proliferation, tumor invasion, and tumor-node-metastasis (TNM) stage [14-20]. Furthermore, LSCC studies showed that reduced PTEN expression is also related to cell proliferation, tumor invasion, lymphatic metastasis, and TNM stage [21-23]. Recent studies have showed that PTEN might be regulated by DJ-1 in several cancers, such as renal cell carcinoma, breast cancer, bladder carcinoma, and ovarian carcinoma [8,24-26]. Kim RH [8] found that DJ-1 could activate cell proliferation and transformation by negatively regulating PTEN expression in breast cancer cells. The above evidence suggests that the DJ-1-induced PTEN down-regulation may be involved in LSCC progression and act as activator of the invasion process in LSCC.

To date, the relationship between DJ-1 and clinicopathological data including patient survival in SSCC have not been revealed. The aim of this study was to investigate the relationship between DJ-1 and clinicopathological data including patient survival.

## Material and methods

### Patients

A total of fifty seven SSCC patients were eligible for this study. 2 and 3 patients were excluded because of insufficient tissue samples and incomplete follow-up data, respectively. 52 subjects with SSCCs and 42 subjects with adjacent non-cancerous tissues were thus examined. These patients underwent surgery in our department from January 1996 to September 2006, and clinical follow-up data were completed. The average observation time for overall survival was 62 months for patients still alive at the time of analysis, and ranged from 7 to 122 months. Twenty-eight patients (53.8%) died during follow-up. Tumor tissues from the resected specimens and adjacent non-cancerous tissues were used as normal control (tumor and adjacent non-cancerous tissues were confirmed by pathologic examination). The tissues used for immunohistochemistry were fixed in 4% polyformaldehyde and embedded in paraffin. All specimens and clinical data in this study were procured, handled, and maintained according to the protocols approved by Institutional Review Board (IRB), and all of the patients who participated in the study provided informed consent.

The principal inclusion criteria were primary squamous cell carcinoma of the supraglottis type only, no history of previous malignant disease, and no history of previous radio- or chemotherapy. The main clinical and pathologic characteristics of the patients are presented in Table 1: 49 (94.2%) were male and with a median age was 59.0 years (ranging from 39–81 years of age). Clinical staging and the anatomic site of the tumors were assessed according to the 6th edition of the Union Internationale Contre Cancer (UICC) tumor-node-metastasis classification of malignant tumors.

**Table 1:** Clinicopathological parameters of the tumor set

		**Number of cases**	**%**
Gender	Male	49	94.2
	Female	3	5.8
Age(y)	≤ 61	25	48.1
	> 61	27	51.9
pT status	T_is_	3	5.8
	T_1_	1	1.9
	T_2_	11	21.2
	T_3_	24	46.1
	T_4a_	12	23.1
	T_4b_	1	1.9
pN status	N_0_	24	46.2
	N_1_	16	30.7
	N_2_	12	23.1
Stage (UICC)	0	3	5.8
	I	1	1.9
	II	6	11.6
	III	24	46.2
	IVA	17	32.6
	IVB	1	1.9
Tumor grade	G_1_	17	32.6
	G_2_	21	40.5
	G_3_	14	26.9

### Immunohistochemical staining

Immunohistochemistry staining was carried out as previously described [2]. DJ-1 polyclonal antibody (Santa Cruz Biotechnology, Santa Cruz, California, USA) and PTEN monoclonal antibody (Cell Signaling Technology, Denver). DJ-1 staining was graded according to the intensity and extent of staining of the epithelium as previously described, and immunostaining of all slides were evaluated in a blinded manner [2].

### Fluorescent immunohistochemistry

To better confirm the cellular location and the relationship between DJ-1 and PTEN in SSCC tissues, fluorescent immunohistochemistry was performed as described previously [27].

### Statistical analysis

Statistical analysis was performed with the SPSS software (SPSS Standard version 13.0, SPSS). The association of DJ-1 protein expression with SSCC patient’s clinico-pathological features and the recorrelation between molecular features detected with each other were by the *χ*2 test or Fischer’s exact test. For survival analysis, we analyzed all SSCC patients by Kaplan-Meier analysis. Log-rank test was used to compare different survival curves. Multivariate survival analysis was performed on all parameters the Cox regression model. *P* < 0.05 was considered to be statistically significant.

## Results

### DJ-1 and PTEN expression in SSCCs and adjacent non-cancerous tissues

**Figure 1:**
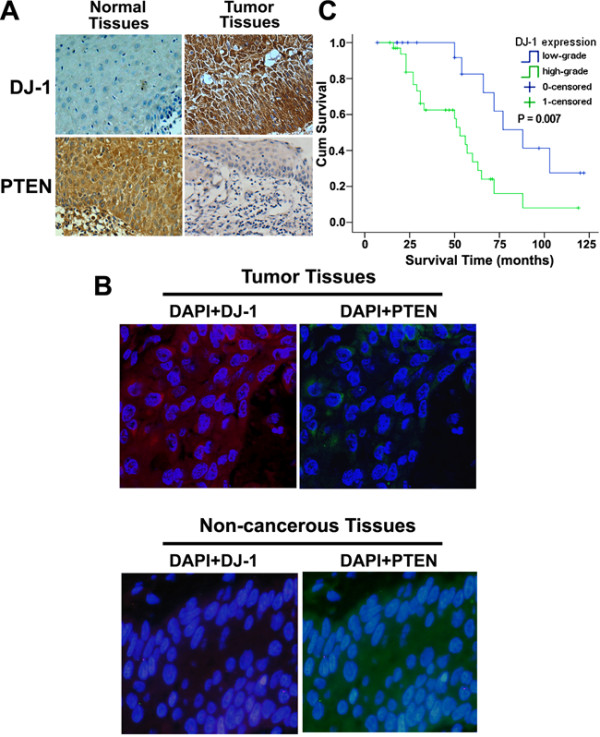
**Expression of DJ**-**1 in SSCC clinical samples and univariate survival analysis. A**. DJ-1 staining showed low expression of DJ-1 in adjacent non-cancerous tissues, and high expression of DJ-1 was found in cytoplasm of SSCC tumor tissues (IHC, 400X). In comparison, PTEN staining of adjacent non-cancerous tissues was stronger and more common than that of SSCCs (IHC, 400X). **B**. Fluorescent-IHC clearly demonstrates that strong expression of DJ-1 is found in cytoplasm of SSCC tumor cells, while poor staining of PTEN was observed in cytoplasm of SSCC tumor cells, and that strong expression of PTEN is found in cytoplasm of adjacent non-cancerous cells, while poor staining of DJ-1 was observed in cytoplasm of adjacent non-cancerous cells (IHC, 400X). **C**. Kaplan-Meier curves with univariate analyses (log-rank) comparing tumors with low- grade DJ-1 expression with those with high-grade DJ-1 expression. Patients with low-grade DJ1 expression had a cumulative 5-year survival rate of 88.0% compared with 53.9% for patients with high-grade DJ-1 expression.

DJ-1 was detected mainly in SSCCs and less frequently in adjacent non-cancerous tissues. In comparison, PTEN staining of adjacent non-cancerous tissues was stronger and more common than that of SSCCs (Figure 1A). To better study the cellular location and the relationship between DJ-1 and PTEN in SSCCs, fluorescent immunohistochemistry was performed, and the results showed that strong expression of DJ-1 is found in cytoplasm of SSCC tumor cells, while poor staining of PTEN was observed in cytoplasm of SSCC tumor cells, and that strong expression of PTEN is found in cytoplasm of adjacent non-cancerous cells, while poor staining of DJ-1 was observed in cytoplasm of adjacent non-cancerous cells (Figure 1B). A summary of DJ-1 and PTEN expression in normal and SSCC tissues is given in Table 2. DJ-1 expression was detected in 88.5% of SSCCs and in 21.0% of adjacent non-cancerous tissues examined, whereas PTEN expression was detected in 46.2% of SSCCs and in 90.5% of adjacent non-cancerous tissues. Moreover, 65.4% of SSCCs were assessed as high grade DJ-1 staining, whereas 78.6% of adjacent non-cancerous tissue had either no or low-grade DJ-1 staining. A significant difference in grade of DJ-1 expression was demonstrated between SSCCs and adjacent non-cancerous tissues (*P* < 0.001). Further more, we find that DJ-1 expression was linked to lymph nodal status (P = 0.042), pT status (P = 0.037), and UICC stage (P = 0.027), and there was no significant association of overall DJ-1 staining intensity with patient age and tumor grading (Table 3).

**Table 2:** **DJ**-**1 and PTEN expression in adjacent non**-**cancerous tissues and SSCCs**

	**DJ**-**1 expression,n (%)**			**PTEN expression,n (%)**			**Total**
	**Absent**	**Low**	**High**	**Absent**	**Low**	**High**	
SSCC	6 (11.5%)	12 (23.1%)	34 (65.4%)	28 (53.8%)	16 (30.8%)	8 (15.4%)	52
Normal	22 (52.4)	11 (26.2%)	9 (21.4%)	4 (9.5%)	10 (23.8%)	28 (66.7%)	42

DJ-1: *χ*2 = 22.917; df = 2; P = 0.000. SSCC, supraglottic squamous cell carcinoma.

PTEN: *χ*2 = 29.769; df = 2; P = 0.000.

**Table 3:** **Relationship between DJ**-**1 expression and various clinicopathological factors**

**Characteristic**	**All cases**	**DJ-1** **Low-grade**	**DJ-1** **High-grade**	**P**
All carcinomas	52	18	34	
Age				1.000
≤ 61	25	9	16	
> 61	27	9	18	
pT status				0.003
T_is-2_	15	10	5	
T_3-4_	37	8	29	
pN status				0.009
N_0_	24	13	11	
N_1-3_	28	5	23	
UICC stage				0.022
0-II	10	7	3	
III-IV	42	11	31	
Histological grade				0.758
G_1_	17	5	12	
G_2-3_	35	13	22	

### DJ-1 is a prognostic marker for SSCC

In univariate survival analysis, cumulative survival curves were calculated according to the Kaplan-Meier method (Table 4). Differences in survival were assessed with the long-rank test. The conventional prognostic parameters pT status, lymph node status, and disease stage according to UICC reached significance for overall survival. DJ-1 positivity was associated with overall survival (P = 0.007). Figure 1C illustrates the impact of DJ-1 expression on survival times.

**Table 4:** **Univariate survival analyses** (**Kaplan**-**Meier**): **survival time of all patients with SSCC according to clinicopathological factors and DJ**-**1 expresion**

**Overall survial**				
**Characteristic**	**No.of cases**	**No.of events**	**5-year survival Rate ( ± SE)**	**P**
DJ-1 expression				0.007
Low-grade	18	7	88.0 ± 8.0	
High-grade	34	21	53.9 ± 5.7	
Age				0.244
≤61	25	11	72.2 ± 7.9	
> 61	27	17	58.5 ± 7.0	
pT status				0.037
T_is-2_	15	5	87.0 ± 10.3	
T_3-4_	37	23	57.5 ± 5.5	
pN status				0.042
N_0_	24	12	76.0 ± 7.7	
N_1-3_	28	16	52.8 ± 5.6	
UICC stage				0.027
0-II	10	3	99.5 ± 8.4	
III-IV	42	25	58.5 ± 5.4	
Histological grade				0.597
G_1_	17	9	68.9 ± 9.4	
G_2-3_	35	19	62.8 ± 6.4	

The multivariate analysis was based on the Cox regression model to test the influence of each parameter on overall survival. We tested the impact of DJ-1 expression on overall survival. The results showed that the overall survival time was significantly dependent on DJ-1 expression, pT status, and UICC stage.

## Discussion

The current TNM staging and histopathological grading systems are useful prognostic indicators for SSCC [3]. However, they have limitations with regard to providing critical information regarding patient prognosis. Patients with the same clinical stage and/or pathological grade of SSCC often display considerable variability in disease recurrence and survival [1,28]. Therefore, new objective measures and biomarkers are necessary to effectively differentiating patients with favorable outcomes from those with less favorable outcomes. Molecular biomarkers in conjunction with standard TNM and histopathological strategies have the potential to predict prognoses more effectively.

DJ-1 protein is coded by exons 27, contains 189 amino acids, and weights about 20 kD, and was firstly defined as an oncogene candidate in 1997 [4]. Recent studies showed that DJ-1 is expressed highly in many types of human malignancies [2,5-15]. Lines of evidence have also suggested that the over-expression of DJ-1 is correlated with more aggressive clinical behaviors of pancreatic, esophageal and lung cancers [10-13]. However, in our recent glottic squamous cell carcinoma study [2], DJ-1 has only been identified as a prognostic marker and activator of cell proliferation, and the expression of DJ-1 was not correlated to clinical lymph node metastasis. This non-invasive role of DJ-1 in glottic squamous cell carcinoma which is contradictory to the invasive role of DJ-1 in other malignancies may be attributed to the clinical and biological behavior of glottic squamous cell carcinoma, as this type of LSCC was poorly invaded in clinic. So, in order to identify whether DJ-1 also play the invasive role in LSCC, SSCC, the more aggressive type of LSCC, was selected in the present study.

Recently, several studies showed that PTEN in human malignancies is associated with cell proliferation, tumor invasion, and TNM stage, and can be down-regulated by DJ-1 in several cancers, such as renal cancer, breast cancer, bladder cancer, and ovarian cancer [8,24-26]. In 2005, Kim RH [8] found that DJ-1 could activate cell proliferation and transformation by negatively regulating PTEN expression in breast cancer cells. In 2012, Lee H [25] showed that over-expression of DJ-1 and loss of PTEN are associated with invasive urothelial carcinoma of urinary bladder. Taken together, we hypothesized that DJ-1 would promote migration and invasion of SSCC via down-regulating the expression of PTEN, and may associated with clinical lymph node status in SSCC.

In the immunohistochemistry-based study, we examined the expression of both DJ-1 and PTEN in SSCC tissue versus adjacent non-cancerous tissue. Our results indicate that the expression of DJ-1 was mainly in SSCCs and less frequently in adjacent non-cancerous tissues, whereas PTEN staining of adjacent non-cancerous tissues was stronger and more common than that of SSCCs (Figure 1A, B). Furthermore, an significant difference in grade of DJ-1 expression was demonstrated between SSCCs and adjacent non-cancerous tissues (*P* < 0.001), and pT status (P = 0.003) and nodal status (P = 0.009) were linked to DJ-1 expression. This scenario is similar to that observed in other type of human cancers [5-13], and the relationship between nodal status and DJ-1 expression in SSCC revealed that DJ-1 may play an invasive role in SSCC. In both univariate and multivariate survival analysis, our study suggests that DJ-1, a prognostic marker for GSCC in our previous study [2], is also a prognostic marker in SSCC (Figure 1C). Thus, expression of DJ-1 appears to have the potential to predict SSCC patients’ outcome.

## Conclusions

In conclusion, to the authors’ knowledge, the current study is the first to demonstrate the relationship between DJ-1 and clinicopathological data including lymph node status in SSCC. Furthermore, survival analysis showed that DJ-1 is an independent prognostic maker for reduced patient survival in SSCC. Collectively, the present findings would provide important information into the future design of individualized therapeutic strategies for SSCC with different DJ-1 expression levels.

## Competing interests

All the authors have no competing interests.

## Authors’ contributions

XLZ performed the experiments and analyzed the data. ZFW and WBL participated in the experiments. HWZ contributed to the acquisition of the data, WJH and YHW has made substantial contribution to collected tissue samples, XLZ and WPW wrote the manuscript, WPW conceived and designed the experiment. All authors have read and approved the final manuscript.
